# Long non-coding RNA KTN1-AS1 promotes progression in pancreatic cancer through regulating microRNA-23b-3p/high-mobility group box 2 axis

**DOI:** 10.18632/aging.203481

**Published:** 2021-08-30

**Authors:** Zhong-Bo Zhang, Ning Liu

**Affiliations:** 1 Department of Pancreatic and Biliary Surgery, The First Affiliated Hospital of China Medical University, Shenyang 110001, Liaoning Province, China

**Keywords:** lncRNA KTN1-AS1, miR-23b-5p, HMGB2, pancreatic cancer, proliferation, invasion

## Abstract

To explore the inhibitory effect of long non-coding RNA (LncRNA) antisense of KTN1 (KTN1-AS1) on the growth of pancreatic cancer (PC) cells by regulating the microRNA-23b-3p (miR-23b-3p)/high-mobility group box 2 (HMGB2) axis. The expression of KTN1-AS1 in tissues and cells was detected by qRT-PCR, and the relationship between KTN1-AS1 and clinicopathological data of patients with PC was analyzed. In addition, stable and transient overexpression and inhibition vectors were established and transfected into PC cells PANC-1, BxPC-3. CCK-8, transwell, and flow cytometry were responsible for the detection of proliferation, invasion, and apoptosis of transfected cells, respectively. The correlation of miR-23b-3p between KTN1-AS1 and HMGB2 was determined by dual luciferase reports, and the relationship between KTN1-AS1 and miR-23b-3p was further verified by RNA immunoprecipitation (RIP). The highly expressed KTN1-AS1 in PC patients was indicative of its high diagnostic value in this disease. Besides, it was found that KTN1-AS1 was linked with the pathological stage, differentiation degree and lymph node metastasis (LNM) of PC patients. Underexpressed KTN1-AS1 led to decreased proliferation and invasion ability of cells and increased apoptosis rate, while the effect of further overexpression of KTN1-AS1 on cells was the opposite. Dual luciferase reporter (DLR) assay confirmed that KTN1-AS1 could target miR-23b-3p, while miR-23b-3p could target HMGB2. Functional analysis showed that the overexpression of miR-23b-3p inhibited the expression of HMGB2 in PC cells and affected cell proliferation, invasion and apoptosis. Co-transfection of Sh-KTN1-AS1 and miR-23b-3p-mimics exhibited that up-regulation of KTN1-AS1 expression could reverse the effect of miR-23b-3p-mimics on PC cells.

## INTRODUCTION

Pancreatic cancer (PC) is a leading cause of tumor-associated mortality [1]. Recently, with advancement of medical technology, significant improvement has been indicated for PC treatment, but due to its high malignancy and easy metastasis, its 5-year survival rate is still under 10% [2, 3]. The absence of obvious symptoms in the early stage of PC and the limitations of routine physical examination partially underlie the poor prognosis of PC patients, leading to the diagnosis of many PC patients in advanced stages [4]. Therefore, searching for the possible pathogenesis of PC and its possible therapeutic targets have important clinical significance for patients with PC. Nevertheless, the regulatory mechanism of PC pathogenesis remains elusive.

Long non-coding RNA (LncRNA) is an RNA of more than 200 bases in length that can regulate transcripts by competitive binding with miRNAs, and has been stated in some reports to be effective in human diseases, especially cancer [5, 6]. For example, it has been reported that LncRNA HOTAIR can regulate HER2 through competitive binding with miR-331-3p, and ultimately accelerate the growth and metastasis of gastric cancer cells [7]. Another case is that, LncRNA ZEB2-AS1 influences growth and metastasis of PC by regulating miR-204/HMGB1 axis [8]. In the present study, we performed RNA-seq analysis in clinical PC samples and identified that the antisense of KTN1, RNA1 (KTN1-AS1), significantly elevated in clinical PC samples relative to para-tumor tissues. KTN1-AS1 is the latest LncRNA that has been found to function as a cancer-promoting factor in several cancers [9–10]. However, the effect of KTN1-AS1 on PC progression remains elusive. We found the interaction between miR-23b-3p and KTN1-AS1 using bioinformatics analysis. MiR-23b-3p induces suppressive function in various cancers. For instance, by targeting cyclin G1, it can regulate progression of osteosarcoma [11]. However, the relationship of KTN1-AS1 with miR-23b-3p in the modulation of PC development is still obscure.

In the present investigation, we explored function and related mechanisms of KTN1-AS1 in PC. We identified a critical role of KTN1-AS1 in promoting PC by targeting miR-23b-3p/HMGB1 axis.

## RESULTS

### High KTN1-AS1 expression in PC

**Figure 1:**
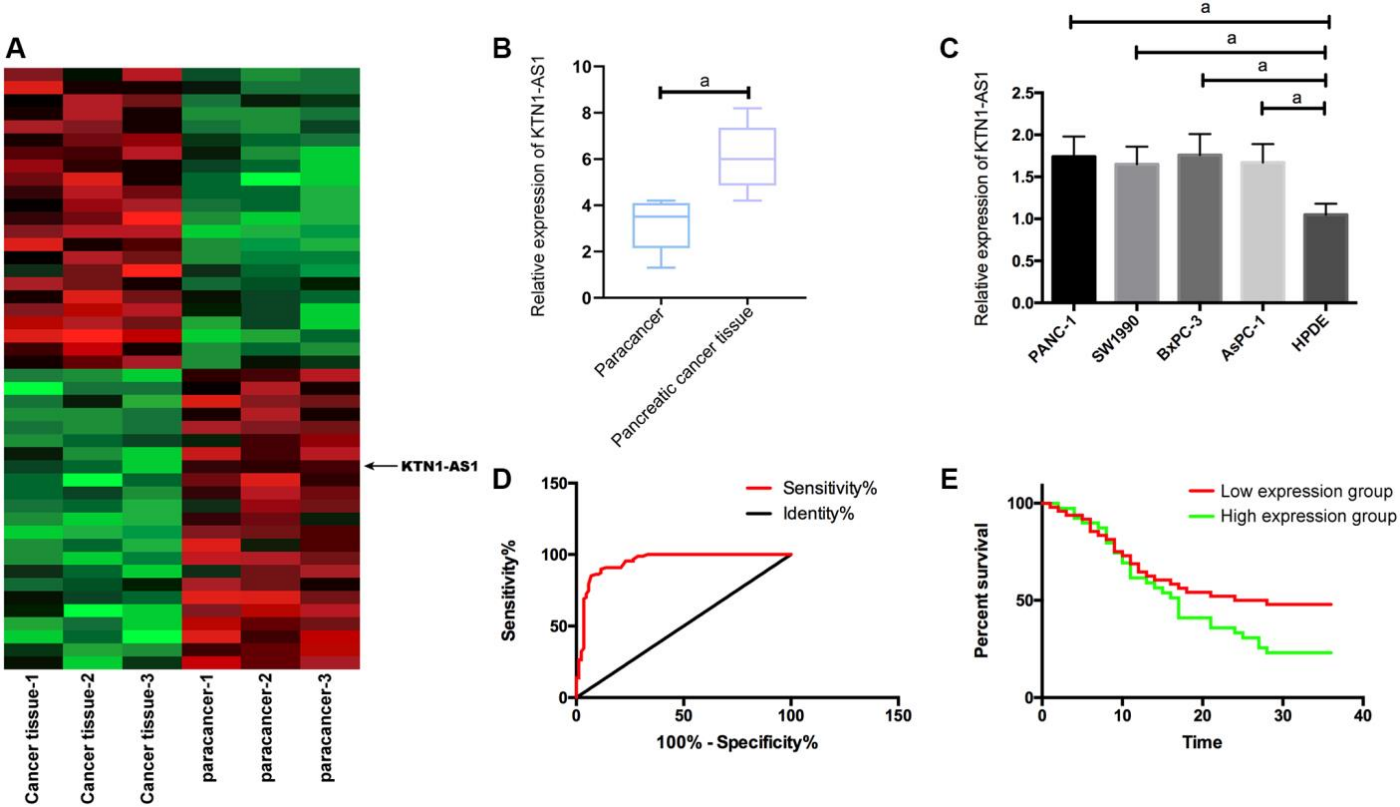
**KTN1-AS1 expression and significance in PC.** (**A**) Heatmap of differentially expressed lncRNAs in the RNA-seq analysis of clinical PC tissue and paracancer tissues, fold change ≥ 2 and *P* value < 0.05. (**B**) KTN1-AS1 expression in PC tissue. (**C**) KTN1-AS1 expression in pancreatic cancer cells. (**D**) ROC curve of KTN1-AS1 in the diagnosis of PC. (**E**) Effects of different KTN1-AS1 expression levels on the survival rate of patients with PC. a indicates *P* < 0.05.

To identify the potential lncRNAs which were involved in the modulation of PC progression, we performed the RNA-seq analysis in clinical PC tissues and adjacent non-tumor tissues. Among the differently expressed lncRNAs, we identified that KTN1-AS1 presented a most significant elevation in the PC tissues (Figure 1A). We then selected KTN1-AS1 for the further analysis. Moreover, quantitative detection of KTN1-AS1 in PC tissues detected by qRT-PCR exhibited that KTN1-AS1 in PC samples was obviously elevated compared with para-tumor tissues, (Figure 1B), and likewise, KTN1-AS1 was observably enhanced in PC cells relative to normal epithelial pancreatic cells. (Figure 1C). The PANC-1 and BxPC-3 cell lines were selected in the subsequent experiments. We demonstrated that the AUC of KTN1-AS1 was 0.946 in the Receiver operating characteristic (ROC) curve (Figure 1D). According to the median expression of KTN1-AS1 (1.68), samples were divided into 48 cases with high KTN1-AS1 expression and 39 cases with low KTN1-AS1 expression. It turned out that KTN1-AS1 was correlated with tumor size, LNM, differentiation degree, and pathological stage. We identified that high KTN1-AS1 expression was correlated with the poor survival rate of the PC patients. (Table 1, Figure 1E).

**Table 1:** Relationship between KTN1-AS1 and pathological data of PC patients.

**Factors**		**lncRNA KTN1-AS1**		***X*^2^ value**	***P* value**
		**High expression (*n* = 56)**	**Low expression (*n* = 44)**		
Age					0.675
	≥59 years old (*n* = 53)	30 (53.57)	23 (52.27)	0.159	
	<59 years old (*n* = 47)	26 (46.43)	21 (47.73)		
Gender					0.957
	Male (*n* = 60)	35 (62.50)	25 (56.82)	0.004	
	Female (*n* = 40)	21 (37.50)	19 (43.18)		
Tumor size					0.776
	≥3cm (*n* = 37)	20 (35.71)	17 (38.64)	0.087	
	<3cm (*n* = 63)	36 (64.29)	27 (61.36)		
TNM staging					<0.001
	Stage I–II	40 (71.43)	13 (29.55)	20.66	
	Stage III	16 (28.57)	31 (70.45)		
Differentiation degree					<0.001
	Low differentiation	32 (57.14)	39 (88.64)	10.57	
	High + moderate differentiation	24 (42.86)	5 (11.36)		
LNM					<0.001
	With (*n* = 41)	15 (26.79)	26 (59.09)	10.38	
	Without (*n* = 59)	41 (73.21)	18 (40.91)		

### KTN1-AS1 induced oncogenetic function in PC cells

**Figure 2:**
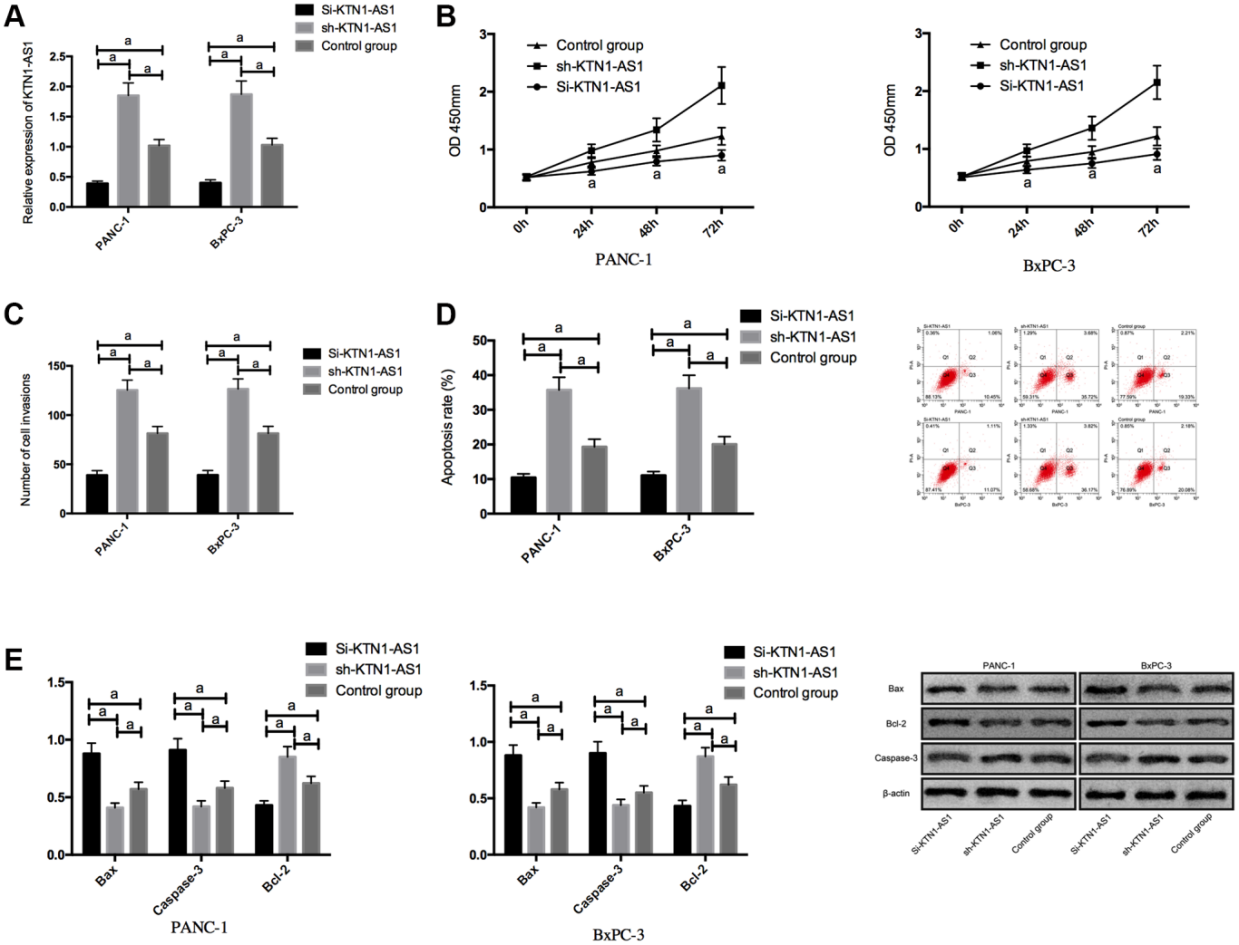
**Effects of KTN1-AS1 on PC cells.** (**A**) Expression of KTN1-AS1 in PC cells after transfection. (**B**) Effects of KTN1-AS1 on the proliferation of PC cells. (**C**) Effects of KTN1-AS1 on the invasion of PC cells. (**D**) Effects of KTN1-AS1 on the apoptosis rate of PC cells. (**E**) Effects of KTN1-AS1 on the apoptosis-related proteins of PC cells. a indicates *P* < 0.05.

We transfected Si-KTN1-AS1, sh-KTN1-AS1 and control NC into PANC-1 and BxPC-3 to observe the effect of KTN1-AS1 on PC cells. It was noticed that the KTN1-AS1 expression in Si-KTN1-AS1 group cells was markedly down-regulated but was increased in Sh-KTN1-AS1 group cells (*P* < 0.05). Knockdown of KTN1-AS1 suppressed the proliferation and invasion of PC cells but accelerated their apoptosis, observably up-regulated pro-apoptotic Bax and Caspase-3 expression, and remarkably down-regulated the anti-apoptotic bcl-2 expression (*P* < 0.05). While on the contrary, KTN1-AS1 could further induce the proliferation/invasion and inhibit apoptosis of PC cells, and meanwhile, markedly declined Bax and Caspase-3 expression, and dramatically enhanced bcl-2 expression. (Figure 2 and Supplementary Figure 1) The expression of Bax, Bcl-2, and caspese-3 was verified by IHC in the clinical PC tissues and paracancer tissues. (Supplementary Figure 2).

### KTN1-AS1 inhibited miR-23b-3p expression by binding to it

To explore the potential downstream miRNAs of KTN1-AS1, we performed the RNA-seq analysis in PANC-1 cells transfected with sicontrol or siKTN1-AS1. Among the differently expressed miRNAs, we identified that miR-23b-3p presented a most significant enhancement by the depletion of KTN1-AS1 in PANC-1 cells (Supplementary Figure 3). We then selected miR-23b-3p for the further analysis. Moreover, we found the potential binding target of KTN1-AS1 and miR-23b-3p. To further verify it, we employed DLR and RIP experiments to confirm their correlation.

**Figure 3:**
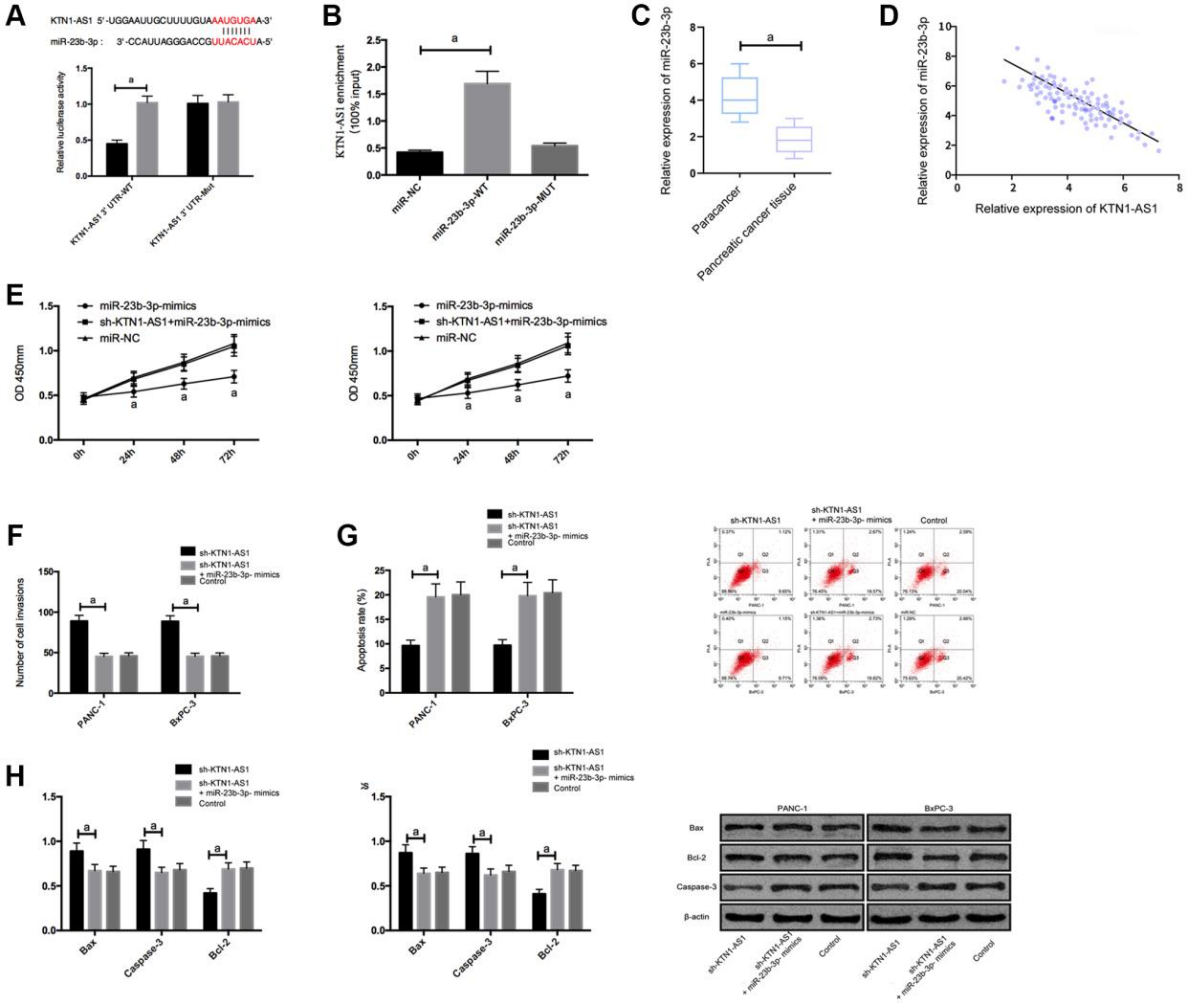
**KTN1-AS1 inhibited its expression by binding to miR-23b-3p**. (**A**) DLR assay. (**B**) RIP experiment. (**C**) MiR-23b-3p expression in PC tissue. (**D**) Correlation analysis between KTN1-AS1 and miR-23b-3p. (**E**) Effects of miR-23b-3p on the proliferation of PC cells. (**F**) Effects of miR-23b-3p on the invasion of PC cells. (**G**) Effects of miR-23b-3p on the apoptosis rate of PC cells. (**H**) Effects of miR-23b-3p on apoptosis-related proteins in PC cells. a indicates *P* < 0.05.

The DLR assay revealed that KTN1-AS1-WT fluorescence activity was markedly inhibited by miR-23b-3p-mimics. RIP experiments showed that the KTN1-AS1 and miR-23b-3p levels interacted with Ago2 were greatly higher than those of IgG. In addition, miR-23b-3p was underexpressed in PC tissues. Correlation analysis indicated a negative relationship of KTN1-AS1 and miR-23b-3p in PC tissues. We then transfected miR-23b-3p-mimics, sh-KTN1-AS1+miR-23b-3p-mimics, and miR-NC, or sh-KTN1-AS1+miR-23b-3p-mimics, and miR-NC to cells. It turned out that after transfecting miR-23b-3p-mimics, the proliferation/invasion/migration of cells was observably inhibited and apoptosis was increased, while the biological function of cells after co-transfection with Sh-KTN1-AS and miR-23b-3p-mimics shown an opposite result. KTN1-AS1 inhibited miR-23b-3p expression by binding to miR-23b-3p, thereby promoting tumor growth. (Figure 3 and Supplementary Figure 1).

### KTN1-AS1 upregulated HMGB2 through competitive binding with miR-23b-3p

**Figure 4:**
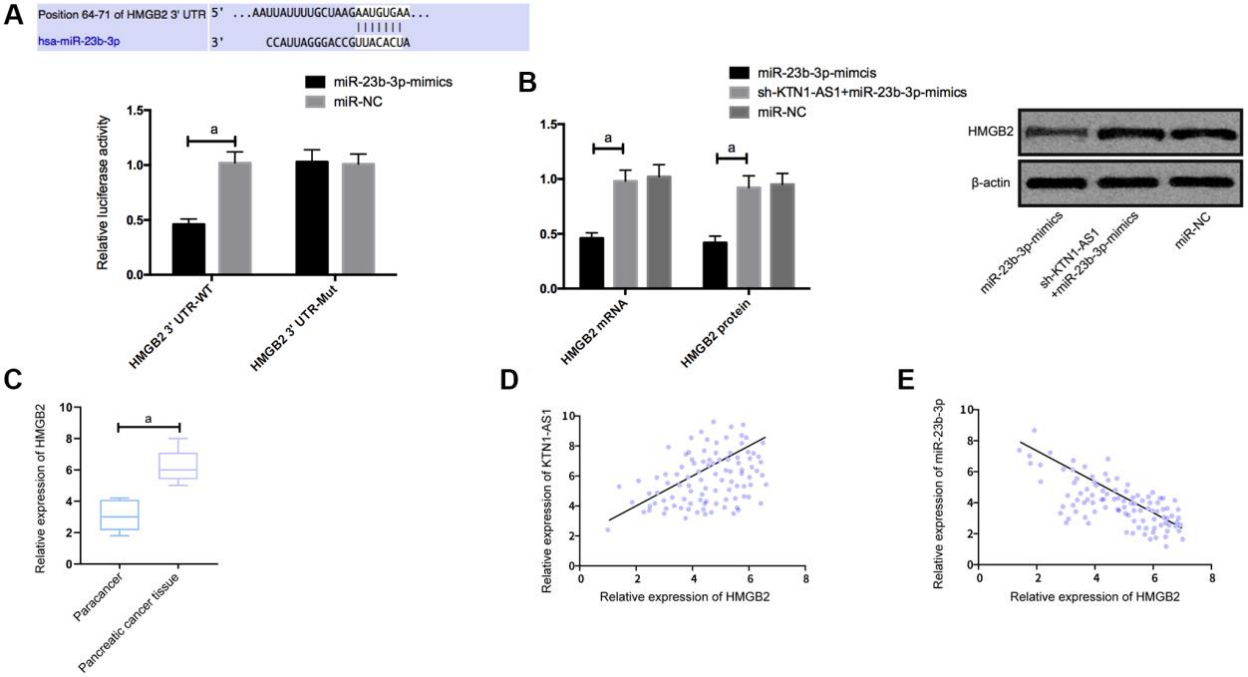
**KTN1-AS1 competitively bound to miR-23b-3p to up-regulate the expression of HMGB2.** (**A**) DLR assay. (**B**) Effects of KTN1-AS1 and miR-23b-3p on HMGB2 protein expression. (**C**) HMGB2 expression in PC tissue. (**D**) Correlation analysis between HMGB2 and KTN1-AS1. (**E**) Correlation analysis between HMGB2 and miR-23b-3p. a indicates *P* < 0.05.

To explore the potential downstream genes of KTN1-AS1 in PC cells, we performed the RNA-seq analysis in PANC-1 cells transfected with sicontrol or siKTN1-AS1. Among the differently expressed genes, we identified that HMGB2 presented a most significant downregulation by the depletion of KTN1-AS1 in PANC-1 cells (Supplementary Figure 3). We then selected HMGB2 for the further analysis. Moreover, we found the targeted binding sites between miR-23b-3p and HMGB2 using Targetscan7.2 online software. The DLR assay exhibited that the luciferase activity of HMGB2 was reduced by miR-23b-3p-mimics. What’s more, HMGB2 was observed to be highly expressed in PC tissues. Correlation analysis revealed a positive relationship of HMGB2 and KTN1-AS1, and a negative relation between HMGB2 and miR-23b-3p. We then transfected miR-NC, miR-23b-3p-mimcis, sh-KTN1-AS1+miR-23b-3p-mimics into cells to detect the expression of HMGB2. It was found that HMGB2 protein and mRNAs expression in the cells were inhibited after upregulation of miR-23b-3p, while the effects were reversed after the co-transfection of sh-KTN1-AS1 and miR-23b-3p-mimics. (Figure 4) The expression levels of HMGB2were verified by IHC in the clinical PC tissues and paracancer tissues. (Supplementary Figure 2).

### Down-regulation of HMGB2 expression inhibited growth and metastasis of PC cells

**Figure 5:**
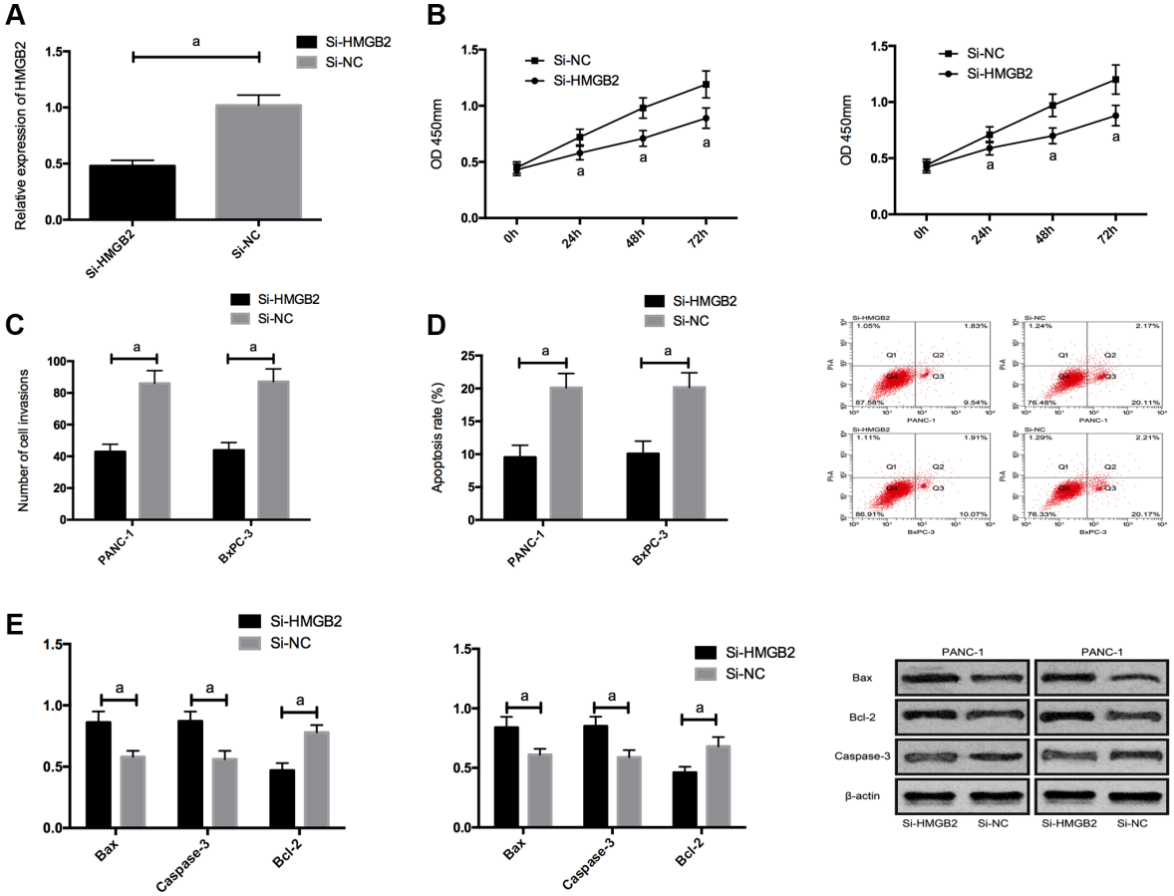
**Effects of HMGB2 on PC cells.** (**A**) HMGB2 expression in PC cells after transfection. (**B**) Effects of HMGB2 on proliferation of PC cells. (**C**) Effects of HMGB2 on invasion of PC cells. (**D**) Effects of HMGB2 on apoptosis rate of PC cells. (**E**) Effects of HMGB2 on apoptosis-related proteins in PC cells. a indicates *P* < 0.05.

For the purpose of further exploring the impact of HMGB2, we silenced HMGB2, and found that proliferation/invasion was suppressed and apoptosis was elevated by Si-HMGB2, and the Bcl-2, N-cadherin, and vimentin expression was declined, and the bax and Caspase-3 expression was boosted. (Figure 5 and Supplementary Figure 1).

### KTN1-AS1-mediated miR-23b-3p/HMGB2 promoted tumor formation in nude mice

**Figure 6:**
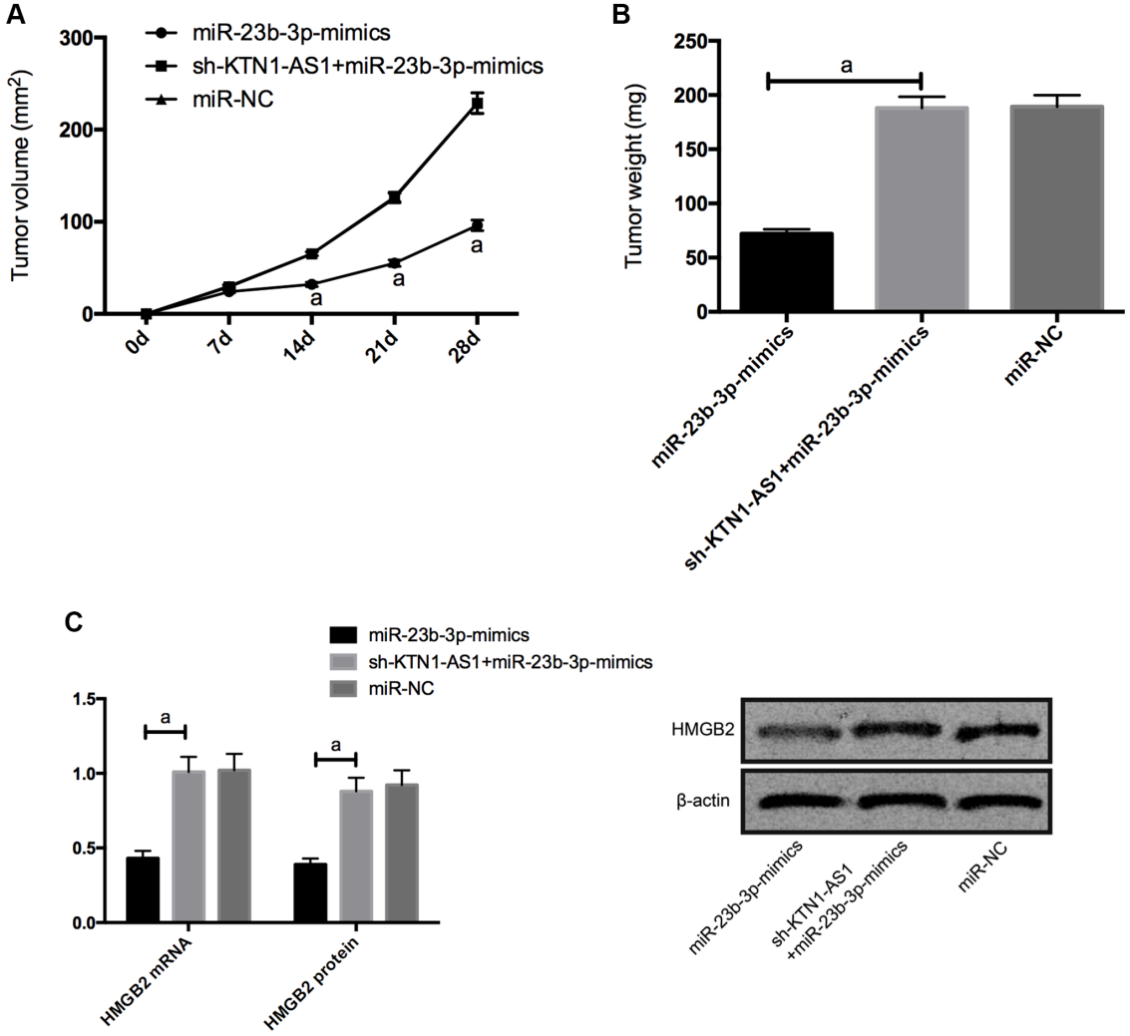
**KTN1-AS1-mediated miR-23b-3p/HMGB2 promoted tumor formation in nude mice.** (**A**) Changes of subcutaneous tumor volume in nude mice within 28 days. (**B**) Tumor volume of nude mice was on the 28th day. (**C**) Relative expression of HMGB2 protein and mRNAs in nude mouse tumor. a indicates *P* < 0.05.

For the sake of observing whether KTN1-AS1 affected solid tumor through miR-23b-3p/HMGB2 axis, we carried out tumorigenesis experiment in nude mice. By injecting miR-NC, miR-23b-3p-mimcis, sh-KTN1-AS1+miR-23b-3p-mimics subcutaneously into nude mice, we found that the tumor size of nude mice injected with miR-23b-3p-mimcis decreased observably, while those of nude mice injected with sh-KTN1-AS1+miR-23b-3p-mimics did not differ notably from those of miR-NC. Further measurement of the expression levels of HMGB2 protein and mRNAs in the tumor tissue of nude mice displayed that the expression levels of HMGB2 protein and mRNAs in the tumor of nude mice injected with miR-23b-3p-mimcis was significantly inhibited, while the expression levels of HMGB2 protein and mRNAs in the tumor of nude mice co-transfected with sh-KTN1-AS1+miR-23b-3p-mimics was reversed. (Figure 6) Meanwhile, we validated that KTN1-AS1 overexpression enhanced the tumor growth and the depletion of KTN1-AS1 or HMGB2 repressed the tumor growth in the nude mice. (Supplementary Figure 4).

## DISCUSSION

As a highly malignant and highly destructive tumor disease, pancreatic cancer (PC) has a very poor prognosis even after treatment [12]. In recent years, emerging evidence has shown that LncRNA plays a extremely important part in the progression of cancer. For example, LncRNA HOTAIR [13] and other lncRNAs have been reported as biomarkers or potential therapeutic targets in a variety of tumors. Another case is that LncRNA MALAT1 [14] has shown to be a key regulator of the metastatic phenotype of lung cancer cells. Apart from that, the role of LncRNA in PC has been discussed extensively in the past. For example, some [15] have revealed that LINC01133 and LINC00205 can be used as prognostic biomarkers of PC. Another research [16] has reported that, by interacting with miR-133, LncRNA MIAT affects the proliferation and metastasis of PC. These reports indicate that LncRNAs widely contribute to the modulation of PC development.

As one of the most recently discovered LncRNAs in recent years, LncRNA KTN1-AS1 has been found to accelerate the progression of colorectal cancer cells [9]. Its role in PC, however, remains poorly understood. Here, KTN1-AS1 was highly expressed in PC tissues and cells, and clinical analysis also showed that KTN1-AS1 was bound up with the clinicopathological stage, differentiation degree, and LNM of patients with PC. In addition, the ROC curve analysis revealed a higher value of KTN1-AS1 in the diagnosis of PC, plus that survival incidence of patients with high KTN1-AS1 expression was also noticeably reduced, indicating that the occurrence and development of PC were closely related to the expression of KTN1-AS1. Moreover, We found for the first time that increasing the expression of KTN1-AS1 could noticeably promote the invasion and proliferation of PC cells and inhibit apoptosis, while on the contrary, inhibited KTN1-AS1 presented a conversed effect, which suggested that KTN1-AS1 played a role in promoting oncogenes in PC. These findings indicate that KTN1-AS1 contributes to the malignant progression and is closely associated with clinical development of KTN1-AS1, provide novel evidence of the important function of LncRNA in the regulation of PC progression. There are still some shortcomings in the current investigation. Although the association of KTN1-AS1 with the clinical characteristics of PC was shown, the diagnostic and prognostic value and clinical significance of KTN1-AS1 should be explored in future investigations by more clinical studies. Meanwhile, we just investigated the effect of KTN1-AS1 on PC in PANC-1 and BxPC-3 cell lines, we will validate our conclusion in other PC cell liens in future investigation. In the past, studies [10] found that KTN1-AS1 also served as an oncogene in hepatocellular carcinoma, which was consistent with the effects of KTN1-AS1 in our research. The specific mechanism of KTN1-AS1 in PC, however, remains elusive.

It is well known that LncRNA can regulate the level of post-transcriptional miRNA by exerting its ceRNA function [17]. In present study, the binding targets of miR-23b-3p and KTN1-AS1 were found through bioinformatics prediction, which was then verified by DLR and RIP experiments. According to past reports, miR-23b-3p was underexpressed in gastric cancer [18] and tongue cancer [19], and likewise, here we discovered the low expression of miR-23b-3p in PC, and through correlation analysis, we identified a negative correlation between KTN1-AS1 and miR-23b-3p. Then we found that the proliferation/invasion of PC cells was notably inhibited and the apoptosis was raised after miR-23b-3p overexpression. Previous literature [20] has supported that miR-23b-3p can hinder the occurrence and progression of PC by directly regulating the ANXA2, which is in line with our findings on miR-23b-3p, but the downstream mechanism of our study is not repeated with this study. In the hope of further studying the effects of KTN1-AS1 and miR-23b-3p on PC cells, we cotransfected sh-KTN1-AS1 and miR-23b-3p-mimics into PC cells. It was observed that sh-KTN1-AS1 reversed the inhibitory effect of miR-23b-3p-mimics on the growth of PC cells and the promotion of apoptosis, which was indicative that up-regulating the expression of KTN1-AS1 can promote the growth of PC by regulating miR-23b-3p. These data indicate that miR-23b-3p is involved in KTN1-AS1-mediated PC progression, presenting the innovative evidence of the tumor suppressive function of miR-23b-3p. Meanwhile, we elucidate the correlation of KTN1-AS1 with miR-23b-3p in the modulation of PC progression. The clinical significance of miR-23b-3p needs to be explored by more investigations.

miRNA is known to affect cell function by regulating its targeted mRNA [21]. High-mobility group B proteins can fine-tune the internal environment of the body by interacting with nucleosomes, transcription factors and histones [22, 23]. Many studies [24, 25] have exhibited that the up-regulation of HMGB is related to the enhanced proliferation, invasion ability and decline of apoptosis of many malignant tumor cells. Other studies [26] have clearly pointed out that HMGB2 can be used as a prognostic indicator for patients with PC. In current study, we predicted the targeting association of HMGB2 with miR-23b-3p, and then verified this targeting relationship by DLR, indicating that KTN1-AS1 might be a molecular sponge for miR-23b-3p to regulate HMGB2 expression. So then we confirmed our hypothesis through experiments, sh-KTN1-AS1 could reverse the inhibitory effect of miR-23b-3p-mimics on HMGB2 expression. Furthermore, the tumor formation experiment in nude mice further confirmed that KTN1-AS1 promoted the growth of PC by targeting miR-23b-3p/HMGB2 axis. Our finding provides new insight into the mechanism by which KTN1-AS1 contributes to PC progression by regulating miR-23b-3p/HMGB2 axis. The miR-23b-3p/HMGB2 axis may just one of the downstream mechanisms underlying KTN1-AS1-mediated PC development and other potential molecules should be explored in the future. And more signaling pathways in which KTN1-AS1 may function will be confirmed by more investigations.

However, there are still some shortcomings in this study. A case is that the downstream signaling pathway of HMGB2 remains elusive. In the follow-up research, we will carry out further basic experiments to provide more data to support our conclusions.

## MATERIALS AND METHODS

### Clinical specimens

After patients’ consents, paired PC tissues and paracancerous tissues were taken from 100 PC patients diagnosed and treated in the First Affiliated Hospital of China Medical University, and the specimens were then frozen in liquid nitrogen tanks for follow-up experiments. Inclusion criteria: Patients pathologically diagnosed as PC. Exclusion criteria: Patients who had received any chemoradiotherapy prior to this study; Patients with other malignancies; Patients with severe hepatorenal dysfunction; Patients with severe infection or immune system disorders. Having been approved by the Medical Ethics Committee of the First Affiliated Hospital of China Medical University, this study was carried out after the consent of all enrolled patients or their families, with written informed consent obtained. The expression levels of HMGB2, Bax, Bcl-2, and caspese-3 were verified by immunohistochemistry (IHC) in the samples.

### Cell culture, passage and transfection

PC cell lines (PANC-1, SW1990, BxPC-3, AsPC-1) and human normal pancreatic duct epithelial cells HPDE were all purchased from the ATCC. The PC cell line was cultured at 37°C with 5% CO_2_ in a medium containing 10% PBS (HyClone, Logan, UT, USA) DMEM (Invitrogen, USA). When digestion finished, the cells were cultured and for passage before transfecting. Since RT-PCR results showed that KTN1-AS1 had the most obvious changes in PANC-1 and BxPC-3, these two were selected for the research objects. MiR-23b-3p-mimics, miR negative control (miR-NC), pcDBA3.1 vector containing targeted inhibition KTN1-AS1 plasmid (si-KTN1-AS1), pcDNA3.1 vector containing KTN1-AS1 plasmid (sh-KTN1-AS1), pcDNA3.1 vector containing targeted inhibition HMGB2 plasmid (si-PBX3), and control sequence (Control) were transfected into cells with Lipofectamine™ 2000 kit, respectively. Strictly abode by the instructions, the operation was carried out. The sequences used were as follows: miR-23b-3p mimics, 5′-ATCACATTGCCAGGGATTACC-3; si-KTN1-AS1, 5′-CTTTCATTTCTGCTATCCC-3′; si-PBX3, 5′-CCGUCAAUUUCGCGGAAUUTT-3′.

### RNA-Seq analysis

The RNA-seq analysis was performed to explore the potential correlation of lncRNAs with pancreatic cancer. Total RNA was extracted from 3 paired pancreatic cancer tissues and paracancer tissues. Meanwhile, the RNA-seq analysis was performed to explore the potential downstream miRNAs and mRNAs of KTN1-AS1. Total RNA was extracted from PANC-1 cells transfected with sicontrol or siKTN1-AS1. The RNA sequencing assay was performed to at KangChen Bio-Tech (Shanghai, China) using Illumina HiSeq 4000 (Illumina, San Diego, CA, USA). The differential expression was analyzed based on *P* value and fold change (fold change ≥ 2 and *P* value < 0.05).

### qRT-PCR detection

RNA was isolated from cells and tissues by using a TRIzol reagent (Invitrogen, USA) in accordance with the protocol, followed by reverse-transcription to cDNA by using PrimeScript Master Mix (Takara, Japan). The levels of RNAs were quantified by using a SYBR Premix Ex Taq Kit (Takara). GAPDH and U6 were used as internal control for normalization of gene expression using the 2^–ΔΔCt^ method. The primer sequences are detailed in Table 2.

**Table 2:** Primer sequences.

**Factors**	**Upstream primer**	**Downstream primer**
miR-23b-3p	5′-GAGCATCACATTGCCAGGG-3′	5′-GTGCAGGGTCCGAGGT-3′
U6	5′-GCTTCGGCAGCACATATACTAAAAT-3′	5′-CGCTTCACGAATTTGCGTGTCAT-3′
KTN1-AS1	5′-ATGCACACTTCTCGGCTAAGAGTC-3′	5′-CTACAATGCCACAAGTGATTCCAGC-3′
HMGB2	5′-GGACCCCAATGCTCCTAAAAGGCC-3′	5′-TGCCCTTGGCACGATATGCAGCA-3′

### Detection of protein expression by western blot

The samples were homogenized by RIPA lysis buffer that contains proteinase inhibitors (Sigma) to extract total proteins. The proteins were quantified by BCA assay kit (Beyotime, China). An equal amount of proteins (30 μg) were divided on SDS-PAGE gel, shifted to NC membranes, blocked by 5% silk milk and hatched with specific primary antibodies against HMGB2 (1: 500), Bax (1: 500), Bcl-2 (1: 500), Caspese-3 (1: 500), and β-Actin (1: 1000) at 4°C overnight. Next day, the protein bands were incubated with specific HRP-conjugated anti-mouse and anti-rabbit secondary antibodies at room temperature for 45 minutes, visualized by using an ECL solution (Millipore, Germany) in a Gel Image system (Bio-Rad, Hercules, CA, USA). HMGB2, Bax, Bcl-2, Caspese-3 and β-Actin antibody were all acquired from Santa Cruz Biotechnology (Santa Cruz, CA, USA).

### Cell proliferation assessed by CCK-8

Cell proliferation was detected according to the instructions of CCK-8 kit (Promega, Madison, WI, USA). Forty-eight hours after transfection, cells were gathered and diluted into 3 × 10^4^cell/mL, and inoculated in 96-well plates, 10 μL CCK8 solution was added to each well and continued to culture in an incubator with 5% CO_2_ at 37°C for 2 h. With the help of a microplate reader, the OD value at 450 nm was finally tested to detect cell proliferation. The experiment was duplicated three times.

### Transwell detection

According to the kit instructions (Reanta Biotechnology Co., Ltd., Beijng, China), cells were collected 24 hours after transfection and inoculated on 24-well plates (3 × 10^4^ cells/well). For cell invasion, cells were suspended in FBS-free medium, and seeded in the upper chambers of Transwell plates (Corning, NY, USA), while the lower chambers were filled with complete medium. The upper chambers were collected after incubation for 48 hours, washed in PBS, fixed in methanol, and dyed with 1% crystal violet. Finally, the cell invasion was observed by a microscope.

### Apoptosis test

On the basis of the instructions of Annexin V-FITC/PI apoptosis kit (Biolab Biotechnology Co., Ltd., Beijing, China), the transfected cells were digested with 0.25% trypsin before rinsing with PBS twice. Then, the cells (1 × 10^6^) were treated with binding buffer, and then AnnexinV-FITC and PI were added successively to incubate for 5 min away from light at room temperature. Finally, apoptosis was detected by FACSVerse flow cytometry (Becton Dickinson Company, United States), and the average value was obtained by repeating the experiment for 3 times.

### Targeting correlation of miR-23b-3p between KTN1-AS1 and HMGB2 determined by DLR gene assay

The wild type (WT) and mutated (MUT) sequences of KTN1-AS1 and the 3′-UTR of HMGB2 were inserted into PmirGLO plasmids. Cells were transfected with the constructed vectors and miR-23b-3p mimics, along with the pRL-TK as internal control. The luciferase activity was determined after 48-hours incubation with a dual luciferase reporter assay system (Promega).

### RNA binding protein immunoprecipitation (RIP experiment)

Magna RIP RNA-binding protein immunoprecipitation kit (Millipore) was used for RIP assay in accordance with manufacturer’s instruction. In short, cells lysates were obtained by using lysis buffer and sonication, then incubated with Ago2 antibody or IgG antibody-conjugated beads for 3 hours at room temperature. The precipitation was washed and eluted, the enriched KTN1-AS1 and miR-23b-3p were analyzed by qRT-PCR assay.

### Tumorigenesis experiment in nude mice

Animal experiments were conducted under the approval of the Animal Research Ethics Committee of the First Affiliated Hospital of China Medical University. 4–6 weeks old male nude mice (Charles River, China) were selected, with 5 in each group. After transfection, 200 mL of PANC-1 cells (3 × 10^6^) were injected subcutaneously into the left side of the back of each nude mouse. Tumor size was measured regularly and calculated using formula 0.52 × tumor length × tumor short diameter^2^. The animals were euthanized 30 d after injection to remove the tumor and measure its volume and weight.

### Statistical analysis

Data were expressed as mean ± SD and analyzed by using SPSS 20.0 software. Comparison between two or multiple groups were determined by student’s *t* test or one-way ANOVA analysis followed by LSD-*t*, respectively. *p* < 0.05 was considered as statistically significant.

## Supplementary Materials

Supplementary Figures

## References

[1] Siegel RL, Miller KD, Jemal A. Cancer Statistics, 2017. CA Cancer J Clin. 2017; 67:7–30. 10.3322/caac.21387 28055103

[2] Zhu H, Li T, Du Y, Li M. Pancreatic cancer: challenges and opportunities. BMC Med. 2018; 16:214. 10.1186/s12916-018-1215-3 30463539 PMC6249728

[3] Rombouts SJ, Vogel JA, van Santvoort HC, van Lienden KP, van Hillegersberg R, Busch OR, Besselink MG, Molenaar IQ. Systematic review of innovative ablative therapies for the treatment of locally advanced pancreatic cancer. Br J Surg. 2015; 102:182–93. 10.1002/bjs.9716 25524417

[4] Hwang JA, Jang KM, Kim SH, Kang TW, Song KD, Cha DI, Ahn S. Integration of different criteria for borderline resectable pancreatic cancer using classification tree analysis: the use of radiological tumour-vascular interface in correlation with surgical and pathological outcomes. Clin Radiol. 2018; 73:321.e1–e10. 10.1016/j.crad.2017.11.001 29221719

[5] Batista PJ, Chang HY. Long noncoding RNAs: cellular address codes in development and disease. Cell. 2013; 152:1298–307. 10.1016/j.cell.2013.02.012 23498938 PMC3651923

[6] Dong P, Xiong Y, Yue J, Hanley SJB, Kobayashi N, Todo Y, Watari H. Long Non-coding RNA NEAT1: A Novel Target for Diagnosis and Therapy in Human Tumors. Front Genet. 2018; 9:471. 10.3389/fgene.2018.00471 30374364 PMC6196292

[7] Liu XH, Sun M, Nie FQ, Ge YB, Zhang EB, Yin DD, Kong R, Xia R, Lu KH, Li JH, De W, Wang KM, Wang ZX. Lnc RNA HOTAIR functions as a competing endogenous RNA to regulate HER2 expression by sponging miR-331-3p in gastric cancer. Mol Cancer. 2014; 13:92. 10.1186/1476-4598-13-92 24775712 PMC4021402

[8] Gao H, Gong N, Ma Z, Miao X, Chen J, Cao Y, Zhang G. LncRNA ZEB2-AS1 promotes pancreatic cancer cell growth and invasion through regulating the miR-204/HMGB1 axis. Int J Biol Macromol. 2018; 116:545–51. 10.1016/j.ijbiomac.2018.05.044 29753015

[9] Cao W, Liu JN, Liu Z, Wang X, Han ZG, Ji T, Chen WT, Zou X. A three-lncRNA signature derived from the Atlas of ncRNA in cancer (TANRIC) database predicts the survival of patients with head and neck squamous cell carcinoma. Oral Oncol. 2017; 65:94–101. 10.1016/j.oraloncology.2016.12.017 28109476

[10] Zhang L, Wang L, Wang Y, Chen T, Liu R, Yang W, Liu Q, Tu K. LncRNA KTN1-AS1 promotes tumor growth of hepatocellular carcinoma by targeting miR-23c/ERBB2IP axis. Biomed Pharmacother. 2019; 109:1140–47. 10.1016/j.biopha.2018.10.105 30551364

[11] Wu B, Xing C, Tao J. Upregulation of microRNA-23b-3p induced by farnesoid X receptor regulates the proliferation and apoptosis of osteosarcoma cells. J Orthop Surg Res. 2019; 14:398. 10.1186/s13018-019-1404-6 31779647 PMC6883581

[12] Spivak-Kroizman TR, Hostetter G, Posner R, Aziz M, Hu C, Demeure MJ, Von Hoff D, Hingorani SR, Palculict TB, Izzo J, Kiriakova GM, Abdelmelek M, Bartholomeusz G, et al. Hypoxia triggers hedgehog-mediated tumor-stromal interactions in pancreatic cancer. Cancer Res. 2013; 73:3235–47. 10.1158/0008-5472.CAN-11-1433 23633488 PMC3782107

[13] Tang Q, Hann SS. HOTAIR: An Oncogenic Long Non-Coding RNA in Human Cancer. Cell Physiol Biochem. 2018; 47:893–913. 10.1159/000490131 29843138

[14] Gong N, Teng X, Li J, Liang XJ. Antisense Oligonucleotide-Conjugated Nanostructure-Targeting lncRNA MALAT1 Inhibits Cancer Metastasis. ACS Appl Mater Interfaces. 2019; 11:37–42. 10.1021/acsami.8b18288 30548064

[15] Giulietti M, Righetti A, Principato G, Piva F. LncRNA co-expression network analysis reveals novel biomarkers for pancreatic cancer. Carcinogenesis. 2018; 39:1016–25. 10.1093/carcin/bgy069 29796634

[16] Li TF, Liu J, Fu SJ. The interaction of long non-coding RNA MIAT and miR-133 play a role in the proliferation and metastasis of pancreatic carcinoma. Biomed Pharmacother. 2018; 104:145–50. 10.1016/j.biopha.2018.05.043 29772434

[17] De Martino M, Forzati F, Marfella M, Pellecchia S, Arra C, Terracciano L, Fusco A, Esposito F. HMGA1P7-pseudogene regulates H19 and Igf2 expression by a competitive endogenous RNA mechanism. Sci Rep. 2016; 6:37622. 10.1038/srep37622 27874091 PMC5118720

[18] Xian X, Tang L, Wu C, Huang L. miR-23b-3p and miR-130a-5p affect cell growth, migration and invasion by targeting CB1R via the Wnt/β-catenin signaling pathway in gastric carcinoma. Onco Targets Ther. 2018; 11:7503–12. 10.2147/OTT.S181706 30498363 PMC6207250

[19] Grossi I, Arici B, Portolani N, De Petro G, Salvi A. Clinical and biological significance of miR-23b and miR-193a in human hepatocellular carcinoma. Oncotarget. 2017; 8:6955–69. 10.18632/oncotarget.14332 28036298 PMC5351682

[20] Wei DM, Dang YW, Feng ZB, Liang L, Zhang L, Tang RX, Chen ZM, Yu Q, Wei YC, Luo DZ, Chen G. Biological Effect and Mechanism of the miR-23b-3p/ANXA2 Axis in Pancreatic Ductal Adenocarcinoma. Cell Physiol Biochem. 2018; 50:823–40. 10.1159/000494468 30355917

[21] Amirkhah R, Schmitz U, Linnebacher M, Wolkenhauer O, Farazmand A. MicroRNA-mRNA interactions in colorectal cancer and their role in tumor progression. Genes Chromosomes Cancer. 2015; 54:129–41. 10.1002/gcc.22231 25620079

[22] Bianchi ME, Beltrame M. Upwardly mobile proteins. Workshop: the role of HMG proteins in chromatin structure, gene expression and neoplasia. EMBO Rep. 2000; 1:109–14. 10.1093/embo-reports/kvd030 11265747 PMC1084262

[23] Bianchi ME, Agresti A. HMG proteins: dynamic players in gene regulation and differentiation. Curr Opin Genet Dev. 2005; 15:496–506. 10.1016/j.gde.2005.08.007 16102963

[24] Liu PL, Tsai JR, Hwang JJ, Chou SH, Cheng YJ, Lin FY, Chen YL, Hung CY, Chen WC, Chen YH, Chong IW. High-mobility group box 1-mediated matrix metalloproteinase-9 expression in non-small cell lung cancer contributes to tumor cell invasiveness. Am J Respir Cell Mol Biol. 2010; 43:530–38. 10.1165/rcmb.2009-0269OC 19933377

[25] Guo ZS, Liu Z, Bartlett DL, Tang D, Lotze MT. Life after death: targeting high mobility group box 1 in emergent cancer therapies. Am J Cancer Res. 2013; 3:1–20. 23359863 PMC3555201

[26] Cai X, Ding H, Liu Y, Pan G, Li Q, Yang Z, Liu W. Expression of HMGB2 indicates worse survival of patients and is required for the maintenance of Warburg effect in pancreatic cancer. Acta Biochim Biophys Sin (Shanghai). 2017; 49:119–27. 10.1093/abbs/gmw124 28069585

