# Supplementary material for: Long non-coding RNA KTN1-AS1 promotes progression in pancreatic cancer through regulating microRNA-23b-3p/high-mobility group box 2 axis
Source: Aging (Albany NY). 2021 Aug 30;13(16):20820–35. doi: 10.18632/aging.203481 (PMC8436926; doi:10.18632/aging.203481)
Supplement: Supplementary Figures [file aging-13-203481-s001.pdf]

SUPPLEMENTARY FIGURES

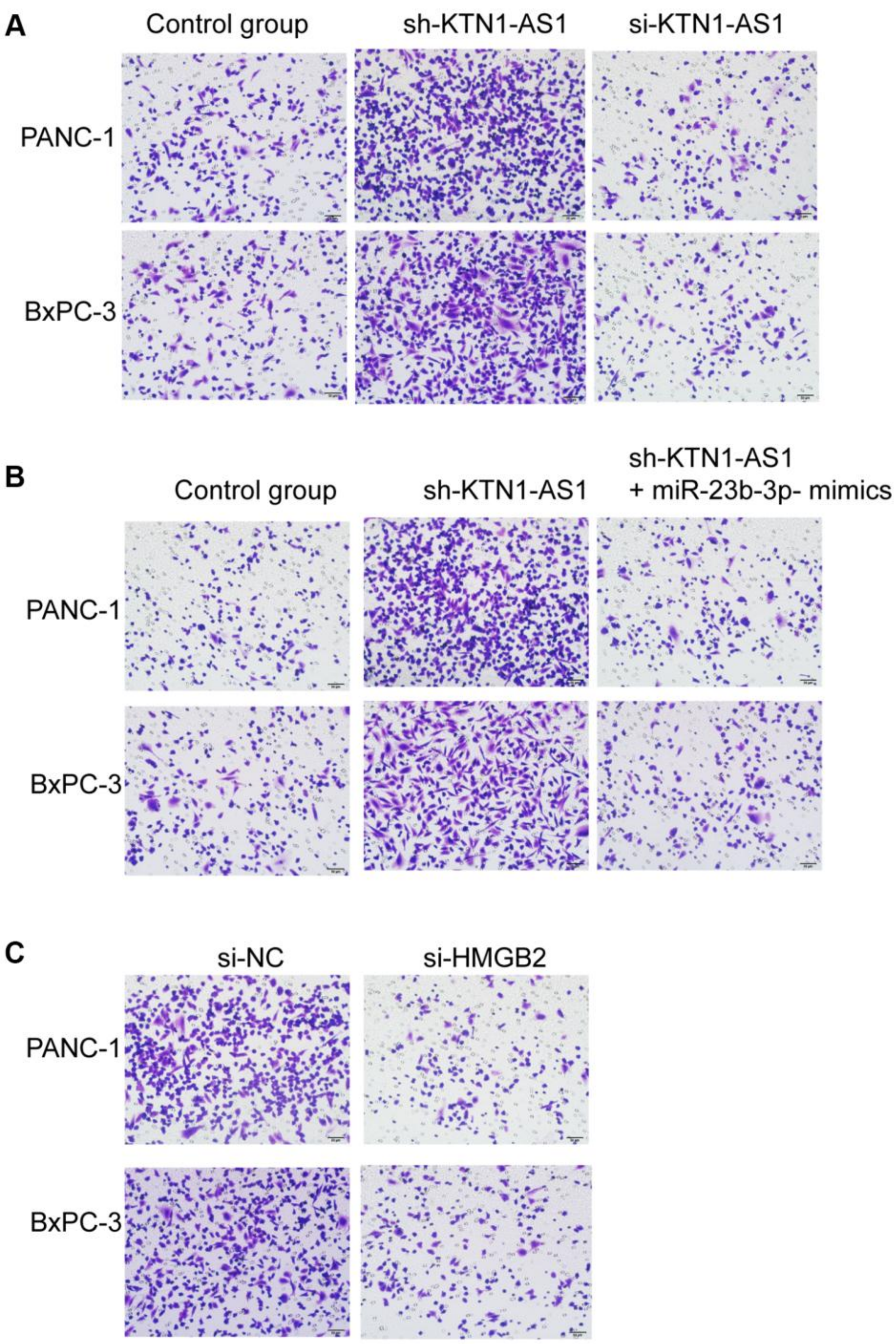

Supplementary Figure 1. The images of the invasion experiments. (**A**) Effects of KTN1-AS1 on the invasion of PC cells, related to Figure 2C. (**B**) Effects of miR-23b-3p on the invasion of PC cells, related to Figure 3F. (**C**) Effects of HMGB2 on the invasion of PC cells, related to Figure 5C.

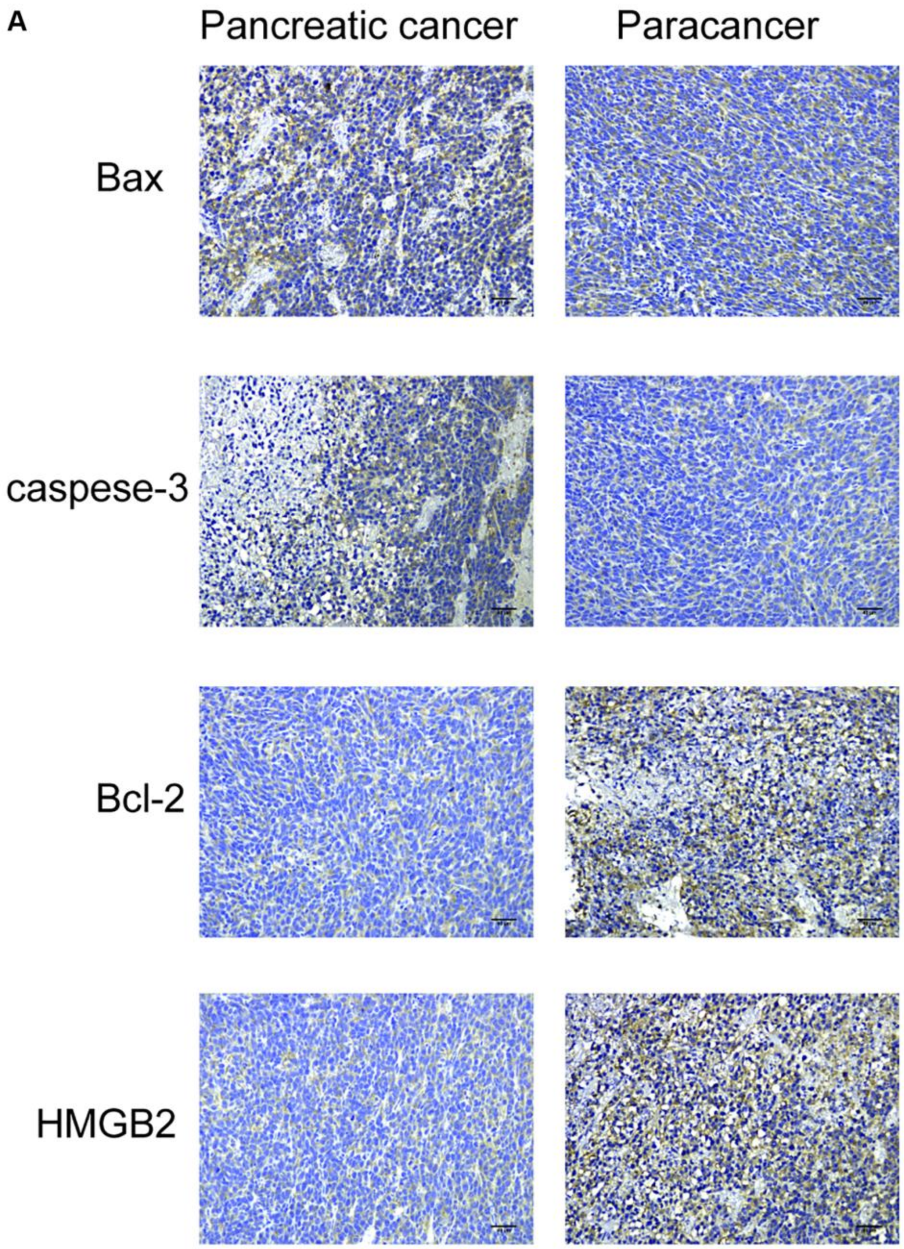

Supplementary Figure 2. The validation of expression levels of HMGB2, Bax, Bcl-2, and caspese-3 in clinical PC tissues. (**A**) The expression levels of HMGB2, Bax, Bcl-2, and caspese-3 were verified by IHC in the clinical PC tissues and paracancer tissues.

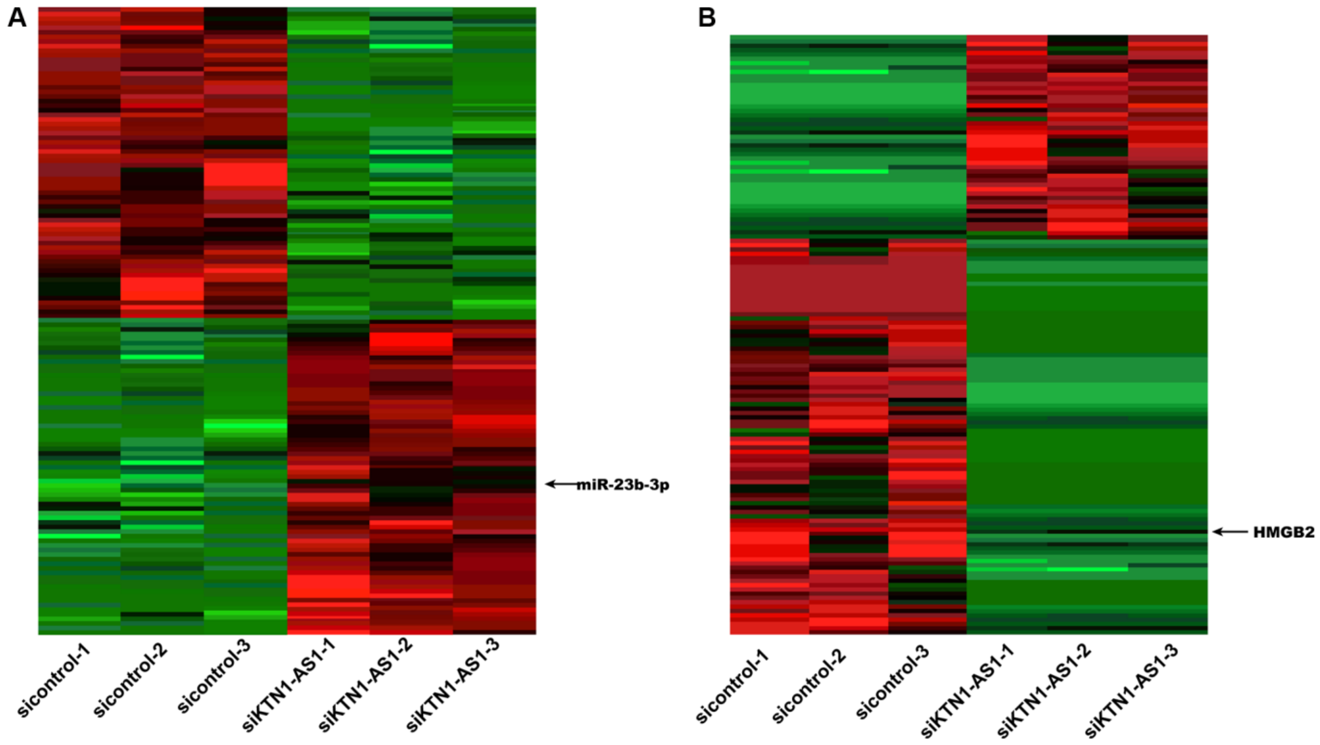

Supplementary Figure 3. The differently expressed miRNAs and genes in KTN1-AS1-depleted PC cells. (**A**) Heatmap of differentially expressed miRNAs in the RNA-seq analysis of PANC-1 transfected with sicontrol or siKTN1-AS1. (**B**) Heatmap of differentially expressed mRNAs in the RNA-seq analysis of PANC-1 transfected with sicontrol or siKTN1-AS1. fold change ≥ 2 and *P* value < 0.05.

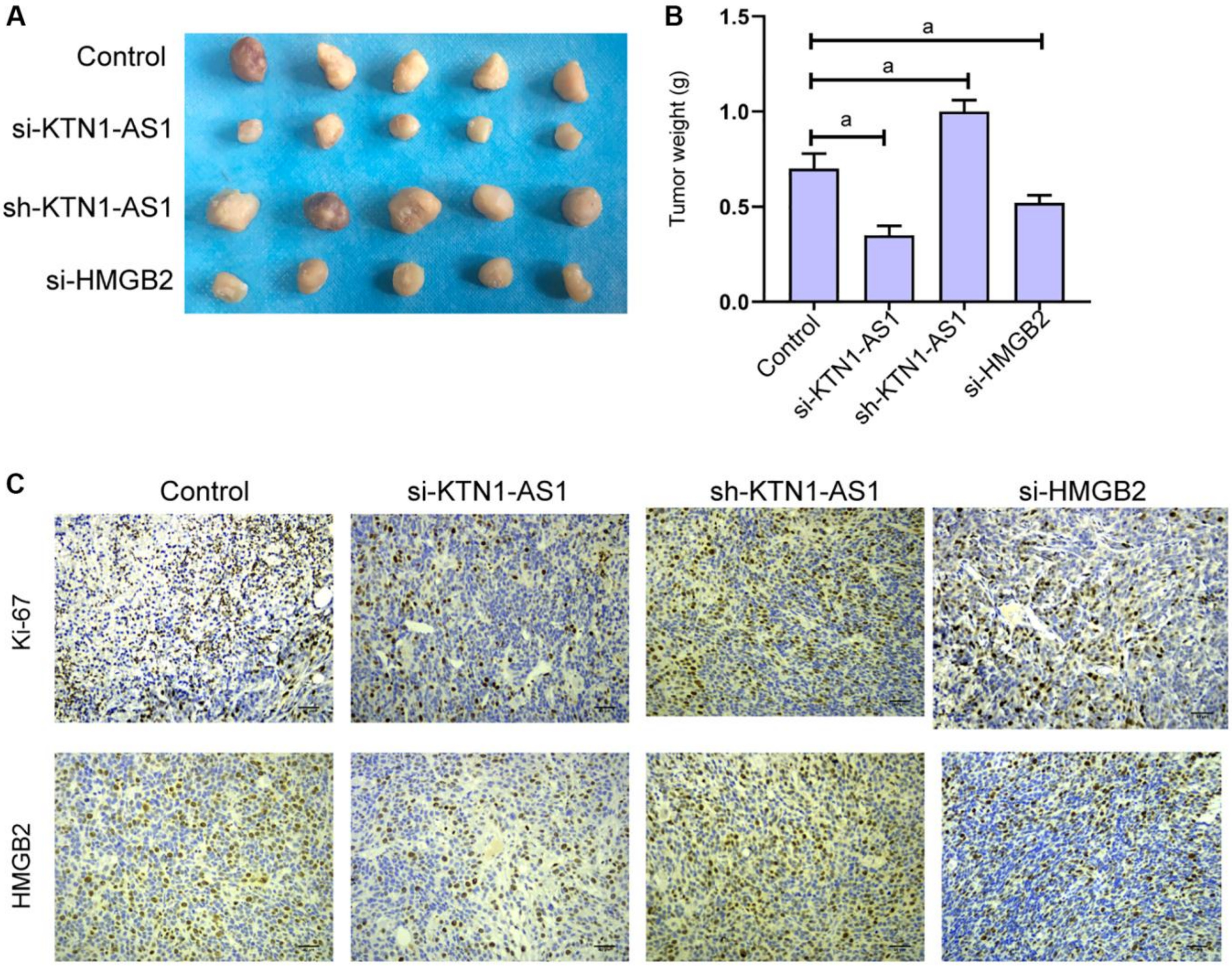

Supplementary Figure 4. The effect of KTN1-AS1 and HMGB2 on tumor formation in nude mice. (**A**) Changes of subcutaneous tumor size in nude mice on the 28th day. (**B**) Tumor weight of nude mice on the 28th day. (**C**) Levels of Ki-67 and HMGB2 and in nude mouse tumor. a indicates $P < 0.05$.
